# Evolution from Monolayers to Two-Dimensional Heterostructures for Enhanced Hydrogen Evolution Reaction: A Theoretical Study

**DOI:** 10.3390/molecules31122176

**Published:** 2026-06-21

**Authors:** Xiaoxiang Hu, Zhiwang Sun, Dongsheng Hu, Jiaan Li, Shifeng Wang

**Affiliations:** Key Laboratory of Plateau Oxygen and Living Environment of Xizang Autonomous Region, College of Science, Xizang University, Lhasa 850000, China

**Keywords:** TMDs, MXenes, electrocatalytic, first-principle calculations, van der Waals heterostructures

## Abstract

Two-dimensional heterostructures have attracted considerable attention in electrocatalytic hydrogen evolution due to their pronounced interfacial effects, tunable electronic properties, and large specific surface areas. In this work, two representative oxygen-terminated transition metal carbides (MXenes) and three typical transition metal dichalcogenides (TMDs) were selected to construct six heterostructures. Using first-principles density functional theory (DFT) calculations, their binding energies, structural stability, electronic structures, and HER catalytic performance were systematically investigated. The results showed that all heterostructures possessed good thermodynamic stability and favorable electronic properties. In particular, SnS_2_/Ti_2_CO_2_, SnSe_2_/Ti_2_CO_2_, SnTe_2_/Ti_2_CO_2_, and SnTe_2_/Zr_2_CO_2_ exhibited near-optimal hydrogen adsorption Gibbs free energy, indicating excellent HER activity. Moreover, the variation in Gibbs free energy of hydrogen adsorption from isolated monolayers to heterostructures could be effectively correlated with the work function difference. The predicted trends provided a useful descriptor for catalytic performance. Overall, this study provides theoretical insights into the rational design of efficient, advanced HER catalysts and contributes to the advancement of sustainable energy conversion technologies. As this work is based solely on first-principles calculations, the predicted catalytic activity of the heterostructure should be regarded as a theoretical prediction and awaits experimental confirmation.

## 1. Introduction

Amid intensifying global energy supply–demand imbalances and escalating ecological and environmental challenges, the development of clean and sustainable energy sources has become an urgent priority. The finite nature of traditional fossil fuels and their high carbon emissions have accelerated the pursuit of renewable alternatives, including solar, wind, and tidal energy [1,2,3]. Hydrogen, as a clean and high-energy-density secondary energy carrier, offers advantages such as zero-emission combustion and versatile feedstock sources. It holds significant potential for applications across transportation, industrial manufacturing, and energy supply systems [4,5,6]. Accordingly, hydrogen energy is increasingly recognized as a cornerstone for constructing low-carbon energy infrastructures, and the exploration of efficient, scalable, and environmentally friendly hydrogen production technologies has emerged as a critical focus of contemporary energy research [7,8].

Currently, industrial hydrogen production primarily includes methods such as fossil fuel-based hydrogen production and water electrolysis [9,10,11]. Compared to traditional fossil fuel-based methods, water electrolysis presents substantial low-carbon benefits. When electricity is sourced from renewable energy such as wind, solar, or hydropower [12,13], it can achieve effectively zero carbon [14,15,16] emissions. Additionally, this technology has a simple process flow and excellent modularity, making it suitable for distributed energy systems [17,18,19,20]. It enables efficient coupling with renewable energy sources while producing high-purity hydrogen, which demonstrates significant application potential.

During the hydrogen evolution reaction (HER) in water electrolysis, electrocatalysts accelerate reaction kinetics and reduce overpotential by modulating the adsorption free energy of hydrogen intermediates (H*) and lowering the activation energy barrier. An ideal HER electrocatalyst should exhibit near-zero hydrogen adsorption free energy (ΔG_H_* ≈ 0) [21,22], high exchange current density, and excellent electrochemical stability [23,24]. Although noble metal Pt and its alloys are widely regarded as benchmark materials for HER performance due to their near-optimal hydrogen adsorption properties, their scarcity and high cost hinder large-scale deployment [25,26,27]. Therefore, developing HER electrocatalysts that combine high activity, low cost, and high stability continues to be a major challenge in water electrolysis for hydrogen production [28,29,30].

Two-dimensional transition metal dichalcogenides (TMDs) [31,32,33] and oxygen-functionalized transition metal carbides (MXenes) [34,35] are highly promising non-precious metal electrocatalysts for the hydrogen evolution reaction (HER). Compared to Pt, these materials offer advantages in Earth abundance, cost-effectiveness, and structural tunability, and their layered structures expose abundant active sites. However, their inherent catalytic activity and electrical conductivity are generally inferior to Pt, leading to higher overpotentials and slower HER kinetics. Specifically, TMDs are often limited by poor charge transport and insufficient stability [36,37], whereas MXenes, despite metallic-level conductivity, face activity limitations due to surface oxidation and terminal functional group dependencies [38,39]. Recent strategies, including defect engineering, heterostructure construction, single-atom anchoring and elemental substitution [40], have proven effective in modulating electronic structures and optimizing hydrogen adsorption free energies, thereby enhancing HER performance toward that of noble metals [41,42]. For example, Szeleszczuk et al. [43] conducted a comprehensive first-principles investigation of the structural, mechanical, electronic, optical, and thermal properties of NaTaO_3−x_S_x_ perovskites. Sulfur substitution at oxygen sites was modeled using the supercell approach. The results revealed that sulfur incorporation induces a structural transition from the cubic to the tetragonal phase and systematically modulates the elastic properties, band gap, and optical response of the material. Shao et al. [44] introduced sulfur vacancies via vacancy engineering to accelerate hydrogen evolution. Chen et al. [45] fabricated MoSe_2_/SnS_2_ heterostructures, improving adsorption at the edges and basal planes of MoSe_2_, thereby enhancing HER kinetics in alkaline media. Furthermore, Hua et al. [46] achieved synergistic optimization of hydrogen and oxygen evolution reactions in a complete water splitting system by constructing a Ti_2_CO_2_/two-dimensional arsenene heterostructure.

The rational construction of TMD/MXene heterostructures has emerged as an effective strategy for significantly improving HER activity, owing to the synergistic effects arising from interfacial interactions, enhanced charge-transfer kinetics, and the increased exposure of catalytically active sites; relevant data are summarized in Table S1. Chen et al. [47] developed a cobalt-doped 1T-MoS_2_/V_2_C MXene heterostructure via an interfacial engineering approach, which significantly promotes the HER kinetics of MoS_2_ by optimizing its electronic structure and interfacial charge-transfer behavior. Asad et al. [48] developed a noble-metal-free Mo-doped ReS_2_@Ti_3_C_2_ MXene(MRT) heterostructure through a facile hydrothermal synthesis route. Benefiting from the synergistic interaction between Mo-doped ReS_2_ and conductive Ti_3_C_2_ MXene, the resulting catalyst exhibited an increased density of exposed active sites and accelerated interfacial charge-transfer kinetics, leading to markedly improved HER activity. Using first-principles density functional theory (DFT) calculations, Wu et al. [49] systematically evaluated the hydrogen evolution reaction (HER) activity of a series of MXenes passivated with transition metal dichalcogenides (TMDs). Their results indicated that all TMD/MXene heterostructures possess outstanding structural stability and high electrical conductivity, both of which are desirable characteristics for efficient electrocatalysis. Notably, Zr_2_CO/VS_2_, Hf_2_CO/VS_2_, and Zr_2_CO/WS_2_ exhibited near-zero hydrogen adsorption free energies (ΔG_H_*) together with excellent stability, highlighting their great promise as next-generation HER electrocatalysts. Hu et al. [50] systematically explored the HER performance of an asymmetric Zr_2_CO/VSe_2_ heterostructure using first-principles density functional theory (DFT) calculations. The calculated hydrogen adsorption Gibbs free energy (ΔG_H_*) was found to be within ±0.1 eV, approaching the thermoneutral adsorption condition. This result suggests highly favorable hydrogen adsorption/desorption kinetics and highlights the considerable potential of the Zr_2_CO/VSe_2_ heterostructure as an efficient HER electrocatalyst.

Inspired by the enhanced catalytic activity of heterostructures in two-dimensional materials, this study constructed six heterostructures by combining three TMDs (SnS_2_, SnSe_2_, SnTe_2_) with two MXenes (Ti_2_CO_2_, Zr_2_CO_2_). SnX_2_ (X = S, Se, and Te) monolayers were chosen as the semiconductor component due to their favorable band-edge positions and relatively small lattice mismatch with MXenes, facilitating the formation of stable van der Waals heterostructures. Among the various MXene candidates, Ti_2_CO_2_ and Zr_2_CO_2_ were selected because they are highly stable oxygen-functionalized MXenes that have been extensively investigated and possess different work functions, enabling effective modulation of interfacial electronic properties. In addition, the chalcogen elements S, Se, and Te, as well as the transition metals Ti and Zr, belong to the same groups of the periodic table but occupy different periods, offering a systematic framework for elucidating the effects of elemental composition on the electronic structure and HER catalytic performance of SnX_2_/Ti_2_CO_2_ and SnX_2_/Zr_2_CO_2_ heterostructures. To the best of our knowledge, this is the first comprehensive DFT study of SnX_2_ (X = S, Se, and Te)/Ti_2_CO_2_ and SnX_2_/Zr_2_CO_2_ MXene heterostructures, providing fundamental insights into their electronic properties and potential catalytic performance for hydrogen evolution reaction (HER) applications. Density functional theory calculations indicate that differences in work function between the two components induce electron transfer at the interface, thereby modulating the catalytic performance of the heterostructures. By comparing the HER performance of the five isolated monolayers with that of the six heterostructures, and analyzing their electronic structures using band structures and work function calculations, we observe a linear relationship between work function difference and the change in hydrogen adsorption Gibbs free energy. In contrast to the majority of previous studies, which rely predominantly on ΔG_H_* as the key activity descriptor, this work examines a preliminary correlation between the work-function difference and HER-related energetic changes. By systematically comparing MXene substrates with different work functions, we identify a previously unexplored trend that establishes a direct correlation between interfacial electronic interactions and HER catalytic performance. These findings provide novel theoretical guidance for designing highly efficient electrocatalysts based on MXenes-derived two-dimensional heterostructures.

## 2. Results and Discussion

### 2.1. Structure and Stability

First, we optimized the monolayer structures of five materials—SnS_2_, SnSe_2_, SnTe_2_, Ti_2_CO_2_, and Zr_2_CO_2_, the structural diagram is shown in Figure S1. To ensure consistency and reliability, as well as the accuracy of the computational methods and models, experimentally synthesized hexagonal isolated monolayers with identical space groups were selected as heterostructure substrates. The lattice constants of the three TMDs monolayers are 3.70, 3.87, 4.12 Å, while those of the two MXenes are 3.03, 3.31 Å, showing good agreement with previous studies [50,51,52,53,54]. Among these, SnX_2_ (X = S, Se, Te) adopts a CdI_2_-type hexagonal layered structure, with a sandwich-like X–Sn–X unit layer stabilized by weak van der Waals interactions. Ti_2_CO_2_ and Zr_2_CO_2_ are oxygen-terminated two-dimensional MXenes derived from their respective MAX phases, exhibiting hexagonal crystal structures with a fundamental unit comprising five atomic layers in an O–Z–C–Z–O (Z = Ti, Zr) sequence. The combination of strong intraplanar covalent bonds and surface-terminated groups endows MXenes with excellent structural stability and tunable electronic properties. Previous studies indicate that most active sites are located on the basal plane [50,53]. Key geometric parameters of the optimized monolayer structures, including symmetry, lattice constants, and bond lengths, are summarized in Table 1.

**Figure 1:**
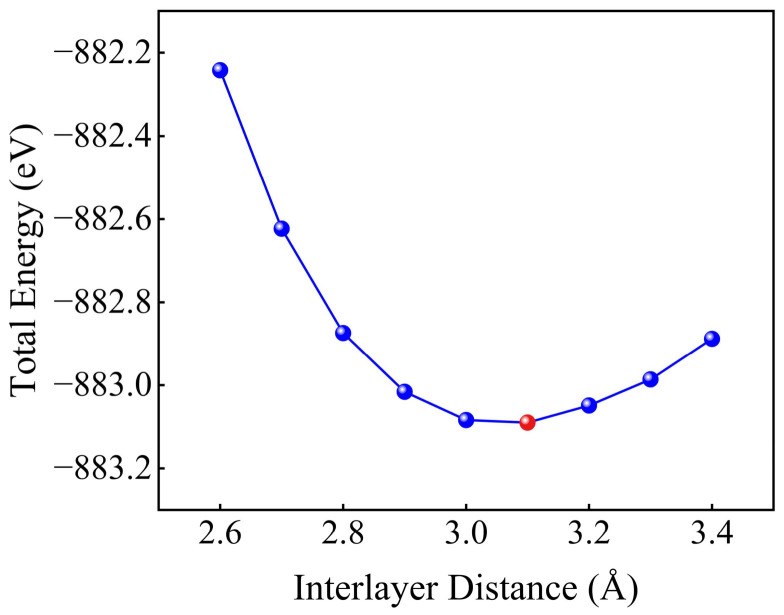
Schematic diagram of total energy variation with interlayer spacing in SnS_2_/Ti_2_CO_2_ heterostructure.

Based on structural optimization calculations for five isolated monolayers, six vertical heterostructures were constructed using three TMDs (SnS_2_, SnSe_2_, SnTe_2_) and two MXenes (Ti_2_CO_2_, Zr_2_CO_2_). To ensure that lattice mismatch remained below 5% for potential experimental realization, $2\sqrt{3}\times 2\sqrt{3}\times 1$ supercell were used for SnS_2_ and SnSe_2_, 3 × 3 × 1 supercell for SnTe_2_, and 4 × 4 × 1 supercells for Ti_2_CO_2_ and Zr_2_CO_2_. The stability of the heterostructures was evaluated through binding energy calculations, defined as:

$$E_{b}=\;\frac{E_{{\mathrm{SnX}}_{2}/Z_{2}{\mathrm{CO}}_{2}}-\;E_{{\mathrm{SnX}}_{2}}-\;E_{Z_{2}{\mathrm{CO}}_{2}}}{A}$$

where $E_{{\mathrm{SnX}}_{2}/Z_{2}{\mathrm{CO}}_{2}}$, $E_{{\mathrm{SnX}}_{2}}$, $E_{Z_{2}{\mathrm{CO}}_{2}}$ represent the total energies of the optimized heterostructure, isolated TMDs monolayer, and isolated MXenes, respectively, and A denotes the surface area of the heterostructure. First, the variation of total energy with interlayer spacing was analyzed for SnS_2_/Ti_2_CO_2_. The results indicate that the total energy reaches a minimum at an interlayer spacing of approximately 3.1 Å, as shown in Figure 1. Further analysis of all six heterostructures (Table 2) shows binding energies ranging from −16.67 to −22.76 meVÅ^−2^, with interlayer distances between 3.04 and 3.24 Å. Here, a more negative binding energy reflects enhanced structural stability and stronger interlayer coupling. This is because a negative binding energy indicates that the total energy of the heterostructure is lower than the combined energies of the constituent isolated monolayers, thereby demonstrating the thermodynamic favorability of heterostructure formation. These values are consistent with typical van der Waals interactions, confirming that SnX_2_ and Z_2_CO_2_ monolayers form stable van der Waals heterostructures.

**Figure 2:**
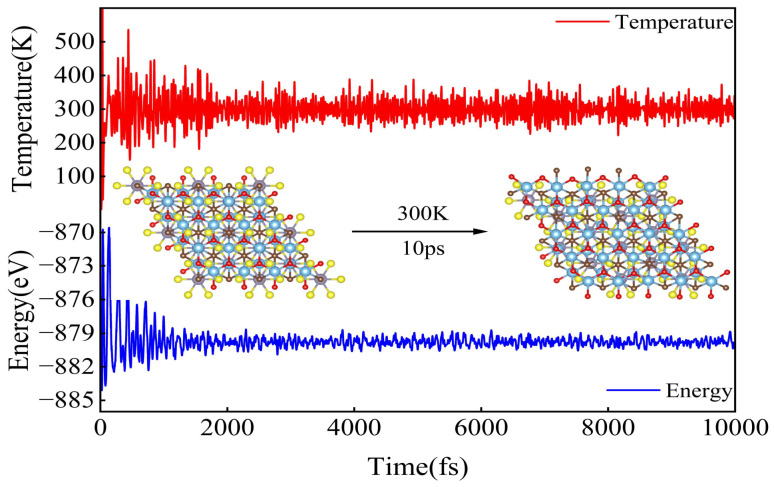
The ab initio molecular dynamics (AIMD) simulates the energy and temperature for SnS_2_/Ti_2_CO_2_.

To evaluate the thermodynamic stability of the constructed heterostructures under environmental conditions, ab initio molecular dynamics (AIMD) simulations were conducted at 300 K for 10 ps. As shown in Figure 2, during the simulation, both the total energy and temperature fluctuated within narrow ranges. No significant structural distortions or bond breakages were observed, indicating that the heterostructure remains structurally stable under room-temperature conditions. Nevertheless, it should be emphasized that a 10 ps AIMD simulation mainly probes short-term thermal fluctuations and is generally regarded as a rapid screening approach for structural integrity rather than a definitive assessment of long-term thermal stability. It is important to emphasize that practical HER conditions involve a complex electrochemical environment, characterized by electrolyte effects, solution pH, and externally applied potentials, which are not incorporated into the current simulations. Consequently, the AIMD results primarily serve as an initial screening of structural robustness and should not be interpreted as definitive proof of long-term stability under actual catalytic operating conditions. The energy and temperature evolutions obtained from ab initio molecular dynamics simulations for the other five heterostructures are presented in Figure S2.

### 2.2. Electronic Properties

**Figure 3:**
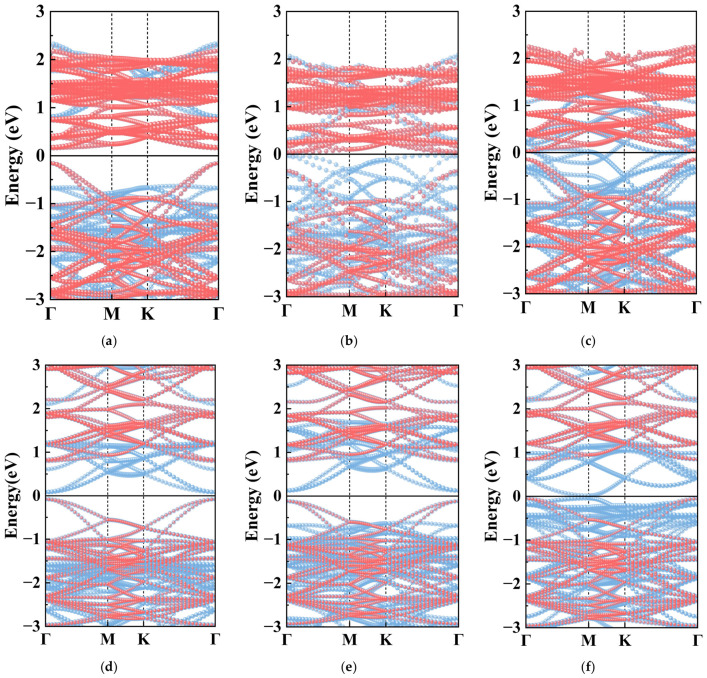
The computed band structures of (**a**) SnS_2_/Ti_2_CO_2_, (**b**) SnSe_2_/Ti_2_CO_2_, (**c**) SnTe_2_/Ti_2_CO_2_, (**d**) SnS_2_/Zr_2_CO_2_, (**e**) SnSe_2_/Zr_2_CO_2_, and (**f**) SnTe_2_/Zr_2_CO_2_. The blue and red lines come from the contribution of SnX_2_ and Z_2_CO_2_ monolayers. The Fermi level was set to zero.

To examine the electronic properties of the individual monolayers and the modifications induced by heterostructure formation, we calculated the band structures of five monolayers (Figure S3) and six corresponding heterostructures (Figure 3). It is well established that the PBE functional tends to underestimate band gaps relative to experimental results and those obtained using hybrid functionals. Consequently, the present study emphasizes the relative variations and trends in the band-gap values, rather than their absolute magnitudes. The results indicate that SnS_2_, SnSe_2_, Ti_2_CO_2_, and Zr_2_CO_2_ are indirect bandgap semiconductors, with bandgaps of 1.57, 0.78, 0.26, and 0.97 eV, respectively, in good agreement with prior theoretical reports. In contrast, SnTe2 exhibits metallic behavior, reflecting its high intrinsic electrical conductivity [50,52,53]. These findings provide a foundation for understanding charge transport and the potential catalytic performance of the constructed heterostructures.

For the constructed heterostructures (Figure 3), SnS_2_/Ti_2_CO_2_, SnSe_2_/Ti_2_CO_2_, SnS_2_/Zr_2_CO_2_, and SnSe_2_/Zr_2_CO_2_ all exhibit semiconducting behavior with band gaps of 0.32, 0.04, 0.16, and 0.25 eV, respectively. Compared with the isolated monolayers, the band gaps of the heterostructures are significantly reduced, and all display direct bandgap characteristics. The demonstrated structural robustness, together with its favorable electronic properties, suggests that the heterostructure is a promising candidate for photoelectrocatalytic applications, particularly for hydrogen evolution reactions. In contrast, due to the intrinsic metallic nature of SnTe_2_, both SnTe_2_/Ti_2_CO_2_ and SnTe_2_/Zr_2_CO_2_ heterostructures retain metallic behavior. The Fermi levels in these metallic heterostructures are primarily dominated by contributions from the SnTe_2_ layer, enabling rapid charge transfer. This enhanced conductivity of the metallic heterostructures is particularly advantageous for facilitating the highly efficient hydrogen evolution reaction (HER).

**Figure 4:**
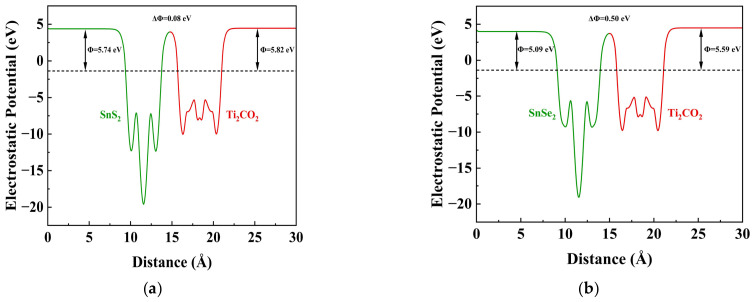

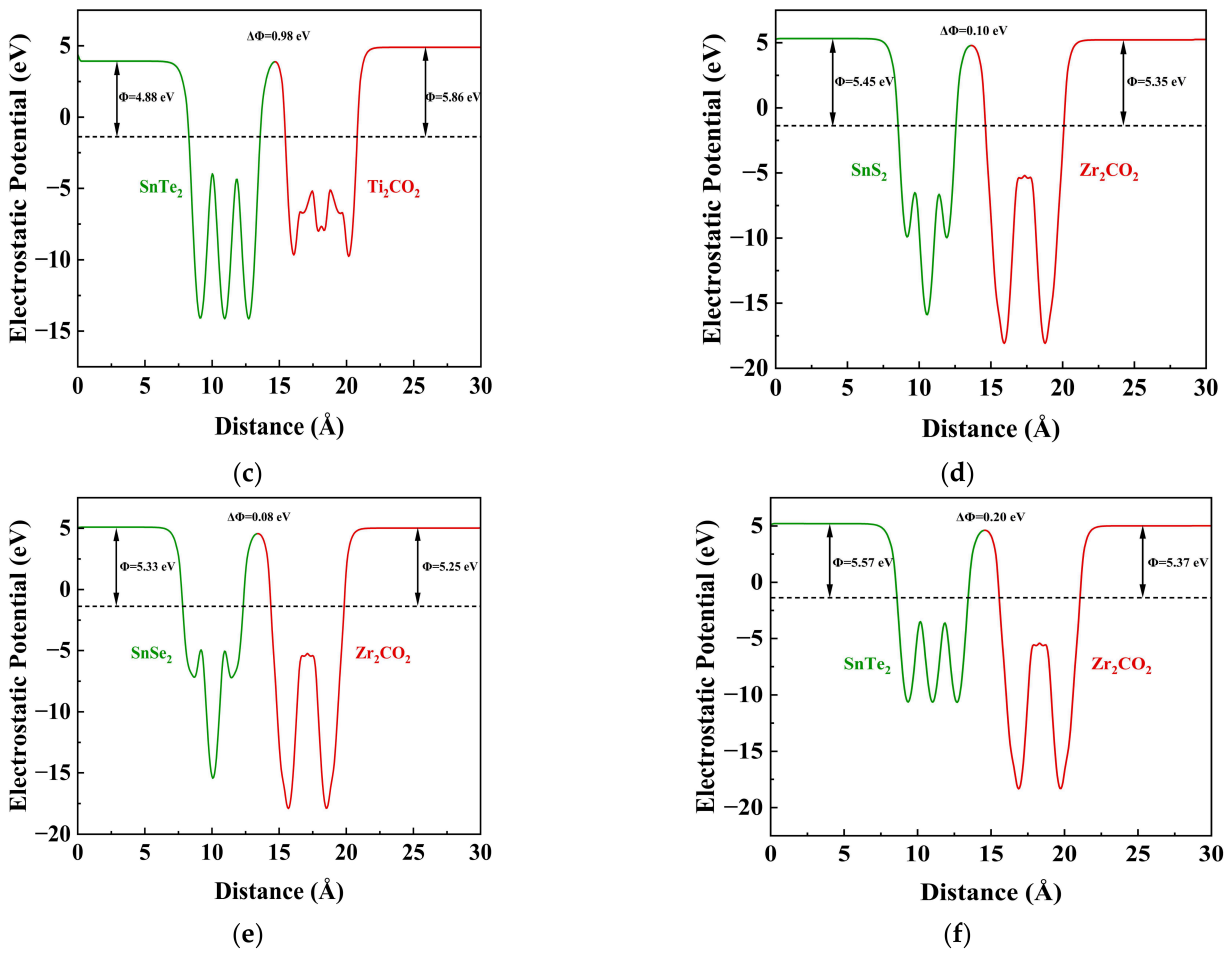
The computed work function of (**a**) SnS_2_/Ti_2_CO_2_, (**b**) SnSe_2_/Ti_2_CO_2_, (**c**) SnTe_2_/Ti_2_CO_2_, (**d**) SnS_2_/Zr_2_CO_2,_ (**e**) SnSe_2_/Zr_2_CO_2_, and (**f**) SnTe_2_/Zr_2_CO_2_.

**Figure 5:**
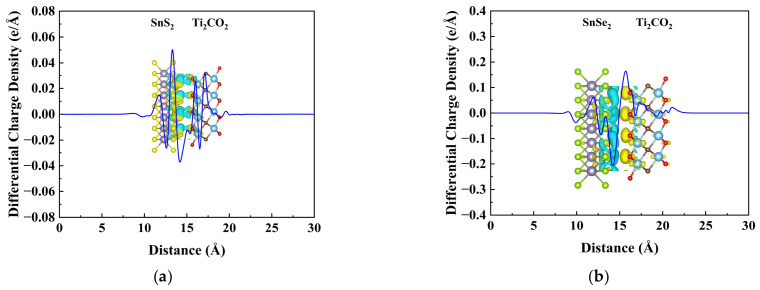

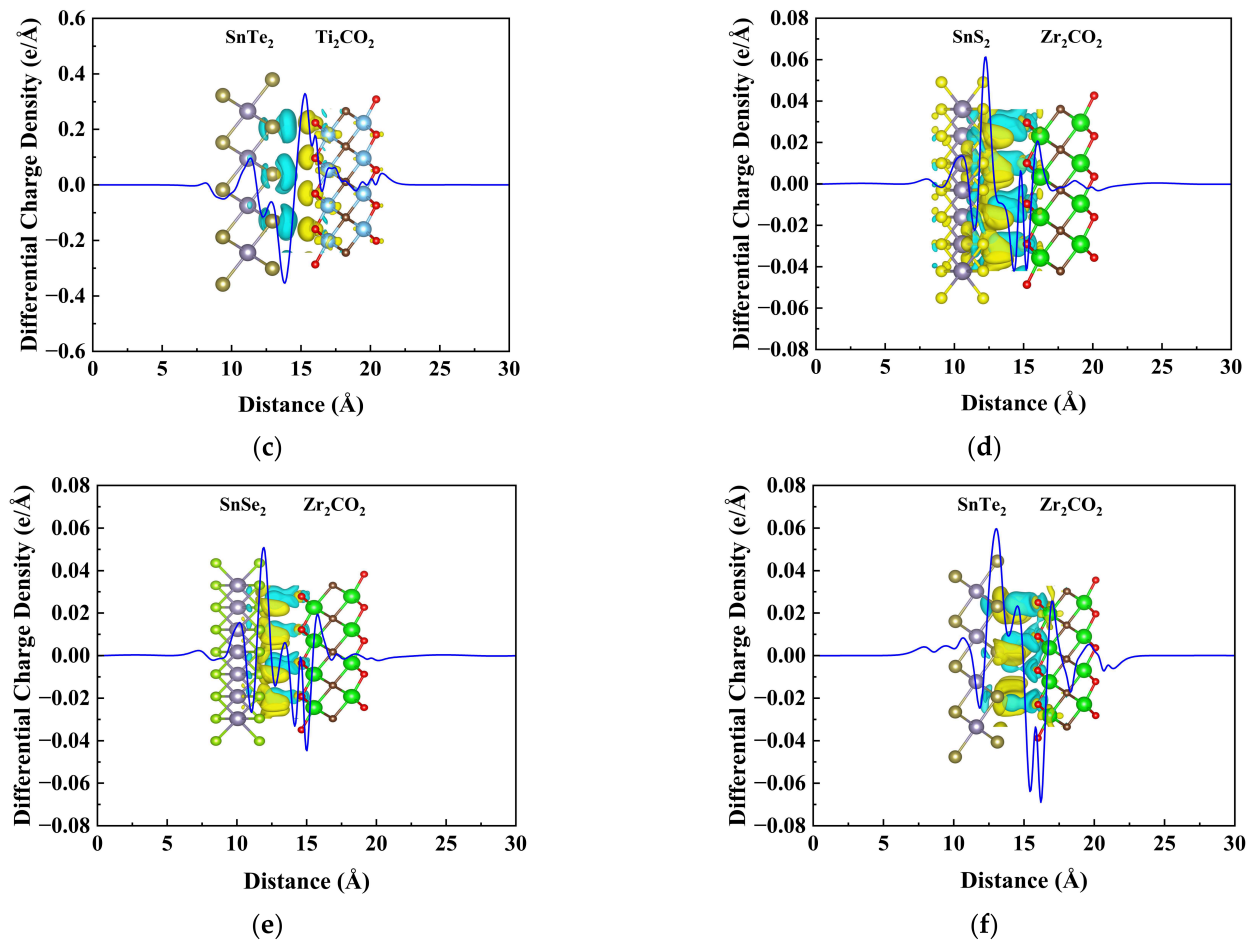
The charge density difference of (**a**) SnS_2_/Ti_2_CO_2_, (**b**) SnSe_2_/Ti_2_CO_2_, (**c**) SnTe_2_/Ti_2_CO_2_, (**d**) SnS_2_/Zr_2_CO_2_, (**e**) SnSe_2_/Zr_2_CO_2_, and (**f**) SnTe_2_/Zr_2_CO_2_.

The work function provides a qualitative measure of charge transfer behavior in heterostructures. We first calculated the work functions of the five monolayers (Table 1), which are 6.57, 5.84, 5.18, 5.83, and 5.05 eV, respectively. These results indicate that SnS_2_ has a relatively high work function, making its surface less susceptible to electron loss, whereas Zr_2_CO_2_ exhibits a lower work function, facilitating easier electron migration when forming heterostructures. Subsequently, the work functions, electrostatic potential distributions (Figure 4), and differential charge densities (Figure 5) of the heterostructures were analyzed. Upon heterostructure formation, the work functions of the constituent materials underwent slight changes, yet significant differences between the two layers persisted. To further elucidate interfacial charge transfer and redistribution, the differential charge density at the heterostructure interface was calculated, providing insight into the electronic interactions that govern charge transport across the interface. The differential charge densities of the *SnX_*2*_/Z_*2*_CO_*2*_ (Z = Ti, Zr)* heterostructure and its individual monolayer components were calculated to investigate interfacial charge redistribution, defined as follows:

$$\rho_{SnX_{2}/Z_{2}CO_{2}}={\rho_{SnX_{2}/Z_{2}CO_{2}}-\rho }_{SnX_{2}}-\rho_{Z_{2}CO_{2}}$$

Among which $\rho_{{\mathrm{SnX}}_{2}/Z_{2}{\mathrm{CO}}_{2}}$,${\;\rho }_{{\mathrm{SnX}}_{2}}$, $\rho_{Z_{2}{\mathrm{CO}}_{2}}$ are the charge densities of the *SnX_*2*_/Z_*2*_CO_*2*_* heterostructure and its single-layer components, respectively. Due to the work function differences, electrons migrate from the material with a lower work function to the one with a higher work function, generating an internal electric field at the interface oriented opposite to the direction of charge migration. Bader charge analysis further quantified the amount of charge transferred across the heterostructure interface. Specifically, SnS_2_, SnSe_2_, and SnTe_2_ transfer 0.10|e|, 0.43|e|, and 0.77|e|, respectively, to Ti_2_CO_2_, while Zr_2_CO_2_ transfers 0.06|e|, 0.08|e|, and 0.11|e|, respectively, to SnS_2_, SnSe_2_, and SnTe_2_. As presented in Table S2, the mismatch in work functions between the constituent materials drives interfacial charge redistribution. Notably, the charge-transfer amount exhibits a clear linear dependence on the work-function difference, with larger disparities resulting in greater electron transfer across the interface. This tunable interfacial charge transfer plays a crucial role in regulating the catalytic activity of the material system.

### 2.3. HER Performance

To further evaluate the hydrogen evolution reaction (HER) performance after heterostructure formation, we calculated the Gibbs free energy of hydrogen adsorption (ΔG_H_*) at the relevant active sites for the five isolated monolayers (Figure S4). The computational hydrogen electrode (CHE) model was employed to evaluate the hydrogen adsorption free energy (ΔG_H_*), which can be expressed as:

ΔG_H_* = ΔE_H_* + ΔG_ZPE_ − TΔS_H_*

where ΔE_H_* denotes the hydrogen adsorption energy at different adsorption sites, while ΔG_ZPE_ and TΔS_H*_ represent the changes in zero-point energy and entropy between adsorbed hydrogen and gaseous hydrogen, respectively. The vibrational energy and entropy of gaseous H_2_ were obtained from the NIST database [55], whereas the zero-point energy and entropy contributions of adsorbed hydrogen were calculated under standard conditions (T = 298.15 K and *p* = 1 bar). The hydrogen adsorption energy was determined according to:

$$\Delta E_{H*}\;=\;E_{H*}\;-\;E_{\mathrm{slab}}\;-\;\frac{1}{2}E_{H_{2}}$$

where E_H*_ and E_slab_ represent the total energies of the catalyst surface with and without hydrogen adsorption, respectively, and E_H_2__ is the total energy of a gas-phase hydrogen molecule.

For SnS_2_, SnSe_2_, and SnTe_2_, the surface S, Se, and Te sites were considered, yielding ΔG_H_* values of 0.87, 0.74, and 0.63 eV, respectively. These results reflect systematic trends; although S, Se, and Te belong to the same group, their outer valence electrons occupy the third, fourth, and fifth electron shells, respectively, leading to progressively weaker interactions with hydrogen and corresponding variations in ΔG_H_*. For the MXenes monolayers Ti_2_CO_2_ and Zr_2_CO_2_, ΔG_H_* was computed for both metal (Ti, Zr) and oxygen surface sites. Ti and Zr sites exhibited high ΔG_H_* values of 2.49 and 2.73 eV, respectively, indicating relatively weak hydrogen adsorption due to the outer valence electrons of these transition metals residing in the fourth and fifth electron shells. In contrast, the O sites displayed markedly different behavior, the ΔG_H_* for O on Ti_2_CO_2_ approaches the ideal value, suggesting excellent catalytic activity for HER, whereas the O sites on Zr_2_CO_2_ showed large positive ΔG_H_* values, indicating weaker hydrogen adsorption. These findings identify Ti_2_CO_2_–O sites as the most promising active centers for efficient hydrogen evolution.

**Figure 6:**
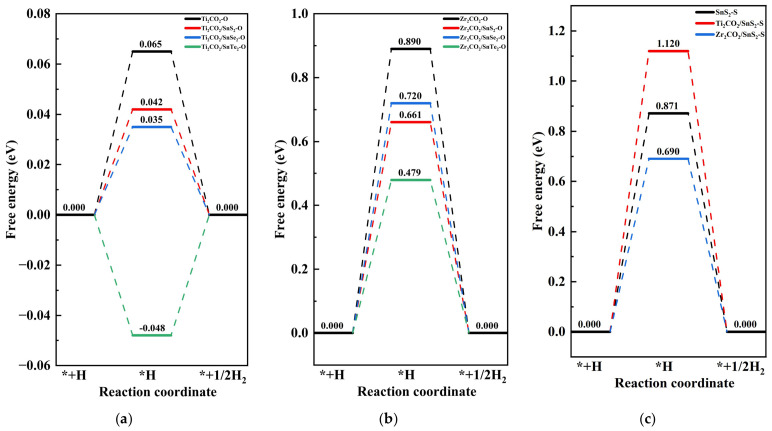

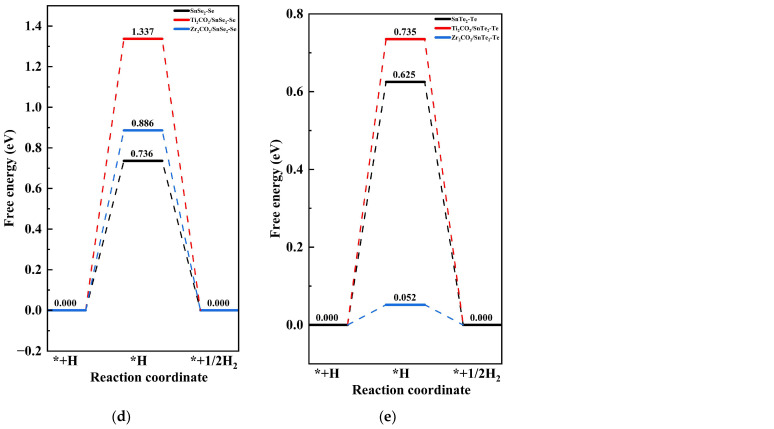
The HER performance change of (**a**) O site on Ti_2_CO_2_, (**b**)O site on Zr_2_CO_2_, (**c**) S site on SnS_2_, (**d**) Se site on SnSe_2_, and (**e**) Te site on SnTe_2_.

After obtaining the hydrogen adsorption Gibbs free energies for the five monolayers, we further examined the effect of heterostructure formation on ΔG_H_* at the same active sites (Figure 6 and Figure S5). For the SnX_2_/Ti_2_CO_2_ heterostructures, ΔG_H_* at the S, Se, and Te sites increased notably, while the Ti site exhibited minimal change, likely due to the structural stability of transition metal centers. In contrast, the O sites showed ΔG_H_* values of 0.042, 0.035, and −0.048 eV for SnS_2_/Ti_2_CO_2_, SnSe_2_/Ti_2_CO_2_, and SnTe_2_/Ti_2_CO_2_, respectively, indicating that interfacial charge transfer significantly enhanced hydrogen adsorption at these sites, promoting HER activity. For the SnX_2_/Zr_2_CO_2_ heterostructures, ΔG_H_* at the S, Se, and Te sites decreased, reflecting charge transfer from Zr_2_CO_2_ to the SnX_2_ layer due to the smaller work function of Zr_2_CO_2_, which strengthened the hydrogen adsorption of the active sites. Simultaneously, ΔG_H_* at Zr and O sites also improved. Notably, the Te site in SnTe_2_/Zr_2_CO_2_ exhibited a ΔG_H_* of 0.05 eV, approaching the ideal value, highlighting its exceptional HER catalytic performance. These results demonstrate that interfacial charge redistribution in heterostructures can effectively tune the adsorption properties of hydrogen and optimize catalytic activity.

To further compare the catalytic activity of different active sites in the heterostructures, the exchange current density [56] was evaluated based on the Gibbs free energy of hydrogen adsorption (ΔG_H_*) according to the following equation:

$$\;\;\;i_{0}=\left\{\begin{array}{c} -ek_{0}\frac{1}{1+e^{\left(-\Delta G_{H^{*}}/k_{b}T\right)}}\;\;\;\;for\;\Delta G_{H^{*}}\leq 0\\ -ek_{0}\frac{1}{1+e^{\left(\Delta G_{H^{*}}/k_{b}T\right)}}\;\;\;\;for\;\Delta G_{H^{*}}\geq 0 \end{array}\right.$$

**Figure 7:**
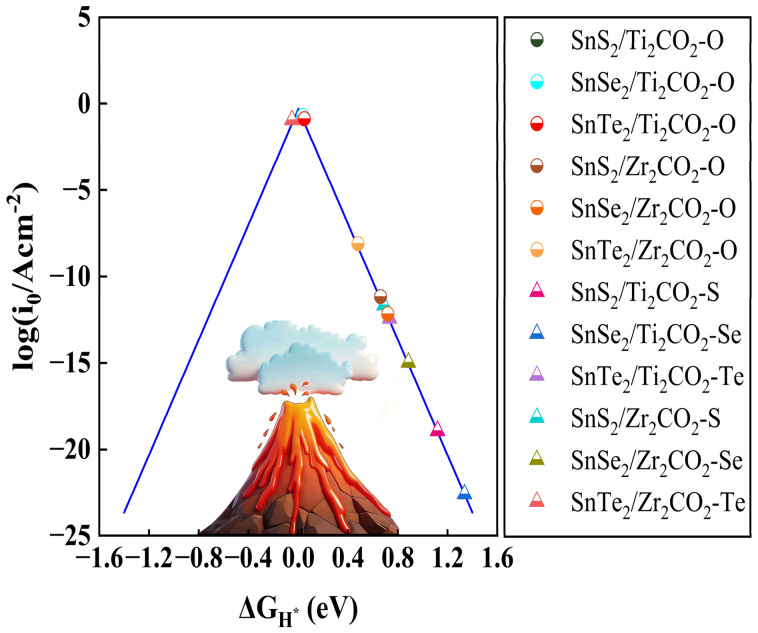
Volcano curve of exchange current (i_0_) as a function of ΔG_H_*.

In this model, *k*_0_ and *k_b_* denote the rate constant and the Boltzmann constant, respectively. The theoretical framework assumes that ΔG_H_* acts as the dominant descriptor determining the reaction kinetics, whereas other complex factors, including activation barriers and proton-transfer processes, are approximated as secondary contributions to the overall catalytic activity. The exchange current density was evaluated from the Gibbs free energy of hydrogen adsorption (ΔG_H_*), and the corresponding results were further visualized in the form of a volcano plot (Figure 7) to facilitate a more intuitive comparison of the catalytic activities. The analysis reveals that the O sites in SnS_2_/Ti_2_CO_2_, SnSe_2_/Ti_2_CO_2_, and SnTe_2_/Ti_2_CO_2_, as well as the Te site in SnTe_2_/Zr_2_CO_2_, exhibit the highest exchange current densities, lying closest to the peak of the volcano curve. These results indicate that these active sites achieve an optimal balance between hydrogen adsorption and desorption, demonstrating HER catalytic performance approaching the ideal.

### 2.4. HER Catalytic Origin

**Figure 8:**
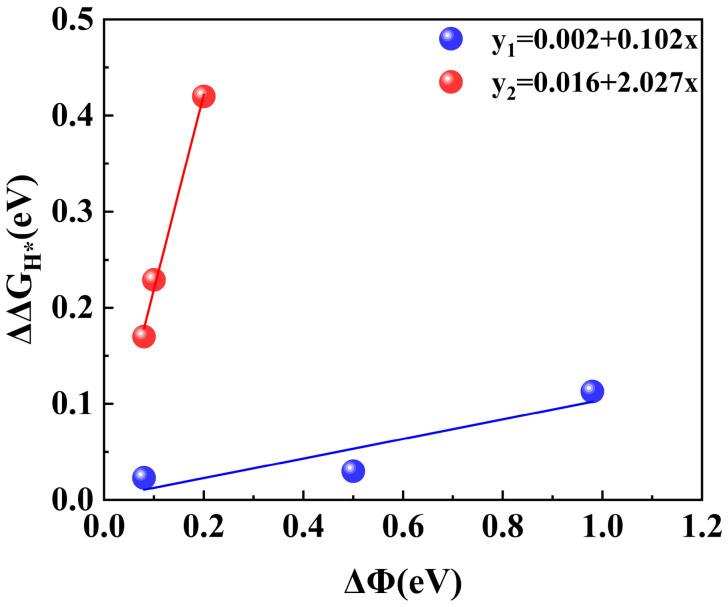
The relationship between work function difference and Gibbs free energy change.

To gain deeper insight into the evolution of HER performance from monolayer to heterostructure systems, we compared the Gibbs free energy of hydrogen adsorption (ΔG_H_*) at the same active sites before and after heterostructure formation. We analyzed the changes in the HER Gibbs free energy at these sites in the monolayer structures of SnS_2_, SnSe_2_, and SnTe_2_, as well as the changes following heterostructure formation with Ti_2_CO_2_ and Zr_2_CO_2_, and correlated these changes with the differences in work functions between the two components (Table S3, Figure 8), where $y_{1}$ represents the Gibbs free energy change of the three monolayers forming a heterostructure with Ti_2_CO_2_, and $y_{2}$ represents the Gibbs free energy change of the three monolayers forming a heterostructure with Zr_2_CO_2_. The analysis reveals a clear linear relationship: larger work function differences lead to more pronounced ΔG_H_* modulation. This trend is consistently observed in both Ti_2_CO_2_ and Zr_2_CO_2_ systems, indicating that within the scope of the limited systems investigated, the work-function difference (ΔΦ) may provide a useful qualitative metric for capturing HER-related energetic trends. Nevertheless, the broader applicability of ΔΦ as a predictive descriptor requires further validation across a wider range of heterostructures and more diverse datasets. Notably, the slopes of the linear correlations differ between the two systems. The more pronounced slope observed for the Zr_2_CO_2_-based systems can be ascribed to its relatively lower work function compared to Ti_2_CO_2_. This difference modulates the interfacial charge redistribution upon heterostructure formation with SnX_2_, thereby altering the electron transfer characteristics at the interface. As a result, the dependence of ΔΔG_H_* on the work function difference (ΔΦ) exhibits distinct trends between the Zr_2_CO_2_ and Ti_2_CO_2_-based systems. This suggests that additional theoretical and experimental studies are needed to fully elucidate the underlying mechanisms.

## 3. Computational Models and Methods

All spin-polarized density functional theory (DFT) calculations were performed using the Vienna Ab initio Simulation Package (VASP) [57,58]. Exchange-correlation effects were described using the Perdew–Burke–Ernzerhof (PBE) functional within the generalized gradient approximation (GGA) [59], while electron–ion interactions were treated via the projected augmented wave (PAW) method [60] with a plane-wave cutoff of 500 eV. To eliminate spurious interactions between periodic images, a vacuum layer of 20 Å was introduced in all slab and heterostructure models. For all asymmetric heterostructures, dipole corrections were applied perpendicular to the surface (along the z axis) to account for the built-in dipole moment and ensure accurate electrostatic potential calculations. To verify the convergence of Brillouin-zone sampling, with all other calculation parameters held constant, Γ-centered [55] 2 × 2 × 1, 3 × 3 × 1, and 4 × 4 × 1 k-point meshes were tested for the heterostructures. As summarized in Table S5, the variation in energy per atom among the three k-point meshes is less than 1 meV, indicating excellent convergence. Therefore, a Γ-centered 2 × 2 × 1 k-point mesh was employed in all subsequent calculations to balance computational efficiency and accuracy. The electronic properties of the heterostructure systems were calculated using a 4 × 4 × 1 k-point mesh for Brillouin-zone sampling. Subsequently, the convergence behavior of the total energy with respect to the plane-wave energy cutoff was examined for the SnS_2_/Ti_2_CO_2_ heterostructure using a 2 × 2 × 1 k-point mesh. The results presented in Table S6 indicate that the total energy becomes well converged at an energy cutoff of 500 eV. Accordingly, a cutoff energy of 500 eV was employed in all subsequent calculations. Structural relaxations proceeded until atomic forces were below 0.02 eV Å^−1^, and the total energy convergence criterion was set to 10^−5^ eV. To accurately capture weak van der Waals interactions in TMDs/MXenes heterostructures, the DFT-D3 method [61] was applied. Crystal structures were visualized using VESTA [62]. Additionally, 10 ps ab initio molecular dynamics (AIMD) [63] simulations were conducted at 300 K to assess the thermodynamic stability of the heterostructures.

Various possible adsorption sites were systematically examined, including the Sn-top, (S/Se/Te)-top, Ti/Zr-top, O-top, and hollow sites. The most stable configurations were determined based on total energy calculations and are presented in the corresponding Figures S6. After obtaining the optimized stable configurations, the hydrogen adsorption coverage at different adsorption sites was evaluated, as summarized in the corresponding Table S4. This strategy effectively eliminates lateral interactions between adsorbates, ensuring the reliability of the calculated adsorption energetics. For hydrogen adsorption at the interface, we initially attempted to define interfacial active sites for the HER calculations. However, it was found that all constructed interfacial models inevitably underwent pronounced structural reconstruction upon geometry optimization, as illustrated in the Figure S7. Consequently, interfacial sites were excluded from subsequent HER activity evaluations. To evaluate the influence of spin polarization, spin-polarized calculations were conducted for the O active sites in all heterostructure systems involved in the hydrogen evolution reaction (HER). As these materials are intrinsically nonmagnetic, the inclusion of spin polarization was found to have a negligible impact on the total energies and electronic band structures. Consequently, non-spin-polarized calculations were adopted throughout this work. The detailed results are presented in Tables S7 and S8.

## 4. Conclusions

In this work, we systematically investigated the structural, electronic, and catalytic properties of SnX_2_/Ti_2_CO_2_ and SnX_2_/Zr_2_CO_2_ heterostructures using first-principles calculations. Constructing heterostructures was found to effectively modulate the catalytic activity of active sites in two-dimensional materials. Notably, the O sites in SnS_2_/Ti_2_CO_2_, SnSe_2_/Ti_2_CO_2_, and SnTe_2_/Ti_2_CO_2_ exhibit excellent hydrogen evolution reaction (HER) activity with ΔG_H_* values approaching the ideal limit, while the Te sites in SnTe_2_/Zr_2_CO_2_ also display near-optimal performance. The observed correlation between ΔΦ and ΔG_H_* is preliminary in nature and should be interpreted as hypothesis-generating rather than conclusive. It suggests a potentially useful trend worthy of further exploration. While ΔΦ demonstrates potential as a qualitative descriptor within the heterostructures investigated herein, its establishment as a generally applicable predictive rule will require systematic validation using substantially larger and more diverse datasets. These findings provide a theoretical framework and guidance for the design and optimization of MXenes-based two-dimensional heterostructure electrocatalysts. Although the theoretical results suggest promising catalytic performance, the present study is purely computational, and experimental investigations are still required to validate the predicted activity of the heterostructure under practical conditions.

## Figures and Tables

**Table 1:** Symmetry, Lattice Parameter, Bond distance, Band Gap and Work Function of five monolayers.

System	Space Group	Lattice Parameter (Å)	Bond Distance (Å)	Band Gap (eV)	Work Function (eV)
SnS_2_	$p\bar{3}m1$	3.70	d_Sn-s_ = 2.60	1.57	6.57
SnSe_2_	$p\bar{3}m1$	3.87	d_Sn-Se_ = 2.75	0.78	5.84
SnTe_2_	$p\bar{3}m1$	4.12	d_Sn-Te_ = 2.98	metallicity	5.18
Ti_2_CO_2_	$p\bar{3}m1$	3.03	d_Ti-C_ = 2.19 d_Ti-O_ = 1.98	0.26	5.83
Zr_2_CO_2_	$p\bar{3}m1$	3.31	d_Zr-C_ = 2.37 d_Zr-O_ = 2.12	0.97	5.05

**Table 2:** Lattice Parameter, Band Gap, Interlayer Distance and Binding energy of six heterostructures.

System	Lattice Parameter (Å)	Band Gap (eV)	Interlayer Distance (Å)	Binding Energy (meV/Å^2^)
SnS_2_/Ti_2_CO_2_	12.24	0.32	3.04	−20.36
SnSe_2_/Ti_2_CO_2_	12.36	0.05	3.04	−22.53
SnTe_2_/Ti_2_CO_2_	12.10	metallicity	3.12	−22.76
SnS_2_/Zr_2_CO_2_	13.10	0.16	3.04	−17.92
SnSe_2_/Zr_2_CO_2_	13.21	0.25	3.12	−18.88
SnTe_2_/Zr_2_CO_2_	13.09	metallicity	3.24	−16.67

## Data Availability

The data presented in this study are available on request from the corresponding authors.

## Supplementary Materials

The following supporting information can be downloaded at: https://www.mdpi.com/article/10.3390/molecules31122176/s1, Table S1: Previously reported TMD/MXene heterostructures for HER electrocatalysis; Table S2: Bader charge data of each constituent in the heterostructure; Table S3: The relationship of ΔΦ and ΔΔGH* (eV); Table S4: Hydrogen coverage associated with different adsorption geometries; Table S5: Convergence of the calculated total energy of SnS_2_/Ti_2_CO_2_ as a function of k-point density; Table S6: Convergence of the total energy of SnS_2_/Ti_2_CO_2_ with respect to the energy cutoff. Table S7: Calculated total energies, zero-point energy (ZPE) corrections, and entropy contributions for different HER active sites. Table S8: Spin-polarization tests of O active sites in all heterostructure systems during HER. Figure S1: (a) Top view of SnS_2_, (b) side view of SnS_2_, (c) top view of Ti_2_CO_2_, (d) side view of Ti_2_CO_2_; Figure S2: The ab initio molecular dynamics (AIMD) simulates the energy and temperature for (a) SnS_2_/Ti_2_CO_2_, (b) SnSe_2_/Ti_2_CO_2_, (c) SnTe_2_/Ti_2_CO_2_, (d) SnS_2_/Zr_2_CO_2_, (e) SnSe_2_/Zr_2_CO_2_, and (f) SnTe_2_/Zr_2_CO_2_; Figure S3: The computed band structures of (a) SnS_2_, (b) SnSe_2_, (c) SnTe_2_, (d) Ti_2_CO_2_, and (e) Zr_2_CO_2_. The Fermi level was set to zero; Figure S4: The HER performance of (a) SnS_2_, SnSe_2_, SnTe_2_, and (b) Ti_2_CO_2_, Zr_2_CO_2_; Figure S5: The HER performance change of (a) Ti site on Ti_2_CO_2_, (b) Zr site on Zr_2_CO_2_; Figure S6: Various possible adsorption sites (a) O site on Ti_2_CO_2_, (b) Ti site on Ti_2_CO_2_, (c) Hollow site on Ti_2_CO_2_, (d) S site on SnS_2_, (e) Sn site on SnS_2_, (f) Hollow site on SnS_2_. Hydrogen atoms are shown in green; Figure S7: Optimization of active sites at the heterostructure interface of (a) SnS_2_/Ti_2_CO_2_, (b) SnSe_2_/Ti_2_CO_2_.
