# Supplementary material for: Evolution from Monolayers to Two-Dimensional Heterostructures for Enhanced Hydrogen Evolution Reaction: A Theoretical Study
Source: Molecules. 2026 Jun 21;31(12):2176. doi: 10.3390/molecules31122176 (PMC13304594; doi:10.3390/molecules31122176)
Supplement: Supplementary file 1 [file molecules-31-02176-s001.zip › molecules-4377195-supplementary.pdf]

Supporting Information

Evolution from Monolayers to Two-Dimensional Heterostructures for Enhanced Hydrogen Evolution Reaction: A Theoretical Study

Table S1. Previously reported TMD/MXene heterostructures for HER electrocatalysis.

Substrate	Heterostructure	ΔG_{H^*} of Substrate(eV)	ΔG_{H^*} of Heterostructure(eV)	Reference
Co-MoS_2	Co-MoS_2/V_2C	-0.29	-0.16	[1]
Ti_3C_2	ReS_2/Ti_3C_2	-0.94	0.65	[2]
Hf_2CO_2	Hf_2CO_2/VS_2	1.19	0.115	[3]
Zr_2CO_2	Zr_2CO_2/VS_2	1.02	-0.194	[3]
Zr_2CO_2	Zr_2CO_2/WS_2	1.02	-0.197	[3]
Hf_2CO_2	Hf_2CO_2/WS_2	1.19	0.19	[3]
Zr_2CO	Zr_2CO/VSe_2	0.14	-0.1-0.1	[4]

Table S2. Bader charge data of each constituent in the heterostructure.

System	monolayer	Bader charges	monolayer	Bader charges	$\Delta\Phi$
SnS_2/Ti_2CO_2	SnS_2	311.89	Ti_2CO_2	384.11	0.08
$SnSe_2$/Ti_2CO_2	$SnSe_2$	311.57	Ti_2CO_2	384.43	0.5
$SnTe_2$/Ti_2CO_2	$SnTe_2$	233.23	Ti_2CO_2	384.77	0.98
SnS_2/Zr_2CO_2	SnS_2	312.06	Zr_2CO_2	639.94	0.1
$SnSe_2$/Zr_2CO_2	$SnSe_2$	312.08	Zr_2CO_2	639.92	0.08
$SnTe_2$/Zr_2CO_2	$SnTe_2$	312.11	Zr_2CO_2	639.89	0.2

Table S3. The relationship of $\Delta\Phi$ and $\Delta\Delta G_{H^*}$ (eV).

System	$\Delta\Phi$ (eV)	$\Delta\Delta G_{H^*}$ (eV)
SnS_2/Ti_2CO_2	0.08	0.023
$SnSe_2/Ti_2CO_2$	0.5	0.03
$SnTe_2/Ti_2CO_2$	0.98	0.113
SnS_2/Zr_2CO_2	0.1	0.229
$SnSe_2/Zr_2CO_2$	0.08	0.17
$SnTe_2/Zr_2CO_2$	0.2	0.411

Table S4. Hydrogen coverage associated with different adsorption geometries.

System	Active site	Surface coverage (ML)
SnS_2	S-top	1/12
$SnSe_2$	Se-top	1/12
$SnTe_2$	Te-top	1/9
Ti_2CO_2	O-top	1/16
Ti_2CO_2	Ti-top	1/16
Zr_2CO_2	O-top	1/16
Zr_2CO_2	Zr-top	1/16

Table S5. Convergence of the calculated total energy of SnS_2/Ti_2CO_2 as a function of k-point density.

K-point density	Energy	Number of	Energy per Atom
1 × 1 × 1	-883.4000	116	-7.6155
2 × 2 × 1	-885.8389	116	-7.6365
3 × 3 × 1	-885.8795	116	-7.6369
4 × 4 × 1	-885.8917	116	-7.6370

Table S6. Convergence of the total energy of SnS_2/Ti_2CO_2 with respect to the energy cutoff.

Energy cutoff (eV)	Energy (eV)	Number of Atoms	Energy per Atom
400	-886.3727	116	-7.6411
450	-885.8953	116	-7.6370
500	-885.8389	116	-7.6365
550	-885.7898	116	-7.6361
600	-885.8692	116	-7.6368

Table S7. Calculated total energies, zero-point energy (ZPE) corrections, and entropy contributions for different HER active sites.

System	E_{Total} (eV)	ZPE (eV)	-TS (eV)
SnS_2/Ti_2CO_2-O	-889.531	0.300	-0.011
SnS_2/Ti_2CO_2-Ti	-887.004	0.187	-0.008
SnS_2/Ti_2CO_2-S	-888.304	0.187	-0.048
$SnSe_2/Ti_2CO_2$-O	-870.208	0.301	-0.011
$SnSe_2/Ti_2CO_2$-Ti	-867.844	0.187	-0.008
$SnSe_2/Ti_2CO_2$-Se	-868.756	0.141	-0.002
$SnTe_2/Ti_2CO_2$-O	-825.980	0.298	-0.012
$SnTe_2/Ti_2CO_2$-Ti	-823.419	0.182	-0.008
$SnTe_2/Ti_2CO_2$-Te	-824.879	0.127	-0.159
SnS_2/Zr_2CO_2-O	-935.818	0.298	-0.012
SnS_2/Zr_2CO_2-Zr	-933.544	0.157	-0.010
SnS_2/Zr_2CO_2-S	-935.678	0.204	-0.029
$SnSe_2/Zr_2CO_2$-O	-921.245	0.298	-0.012
$SnSe_2/Zr_2CO_2$-Zr	-919.066	0.160	-0.010
$SnSe_2/Zr_2CO_2$-Se	-920.921	0.173	-0.044
$SnTe_2/Zr_2CO_2$-O	-869.337	0.298	-0.012
$SnTe_2/Zr_2CO_2$-Zr	-866.916	0.156	-0.011
$SnTe_2/Zr_2CO_2$-Te	-869.591	0.156	0.044
H_2	-6.77	0.28	-0.40

Table S8. Spin-polarization tests of O active sites in all heterostructure systems during HER.

System	E_{Total} (eV)	ZPE (eV)	-TS (eV)
SnS_2/Ti_2CO_2-O	-889.531	0.300	-0.011
$SnSe_2/Ti_2CO_2$-O	-870.207	0.301	-0.011
$SnTe_2/Ti_2CO_2$-O	-825.981	0.298	-0.012
SnS_2/Zr_2CO_2-O	-935.818	0.298	-0.012
$SnSe_2/Zr_2CO_2$-O	-921.245	0.298	-0.012
$SnTe_2/Zr_2CO_2$-O	-869.307	0.297	-0.012

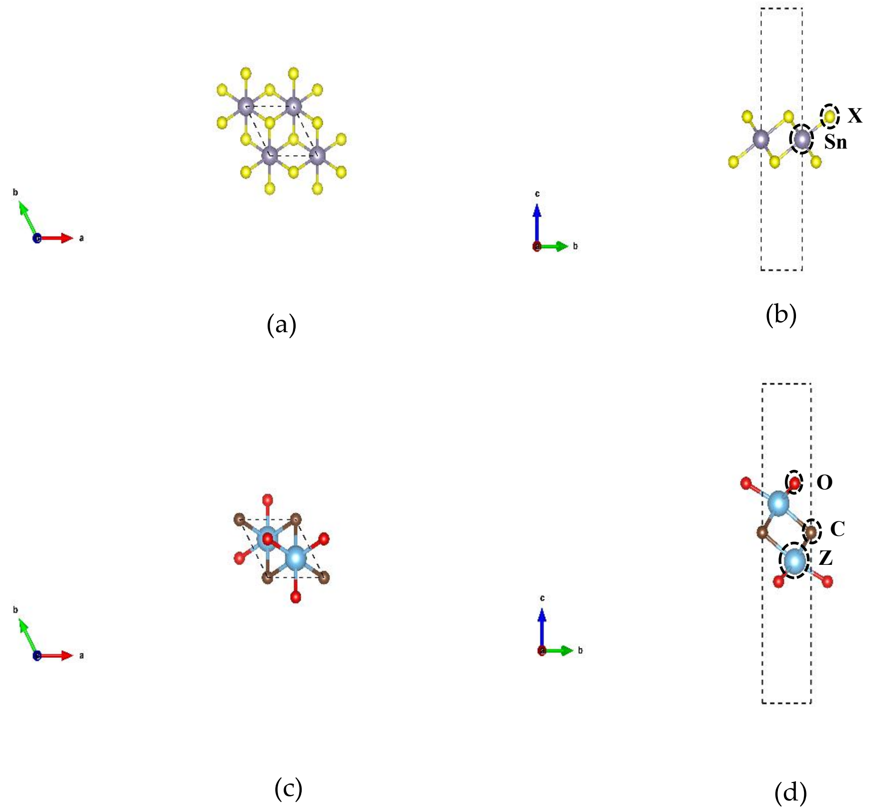

Fig S1 (a) Top view of SnS_2, (b) side view of SnS_2, (c) top view of Ti_2CO_2, (d) side view of Ti_2CO_2.

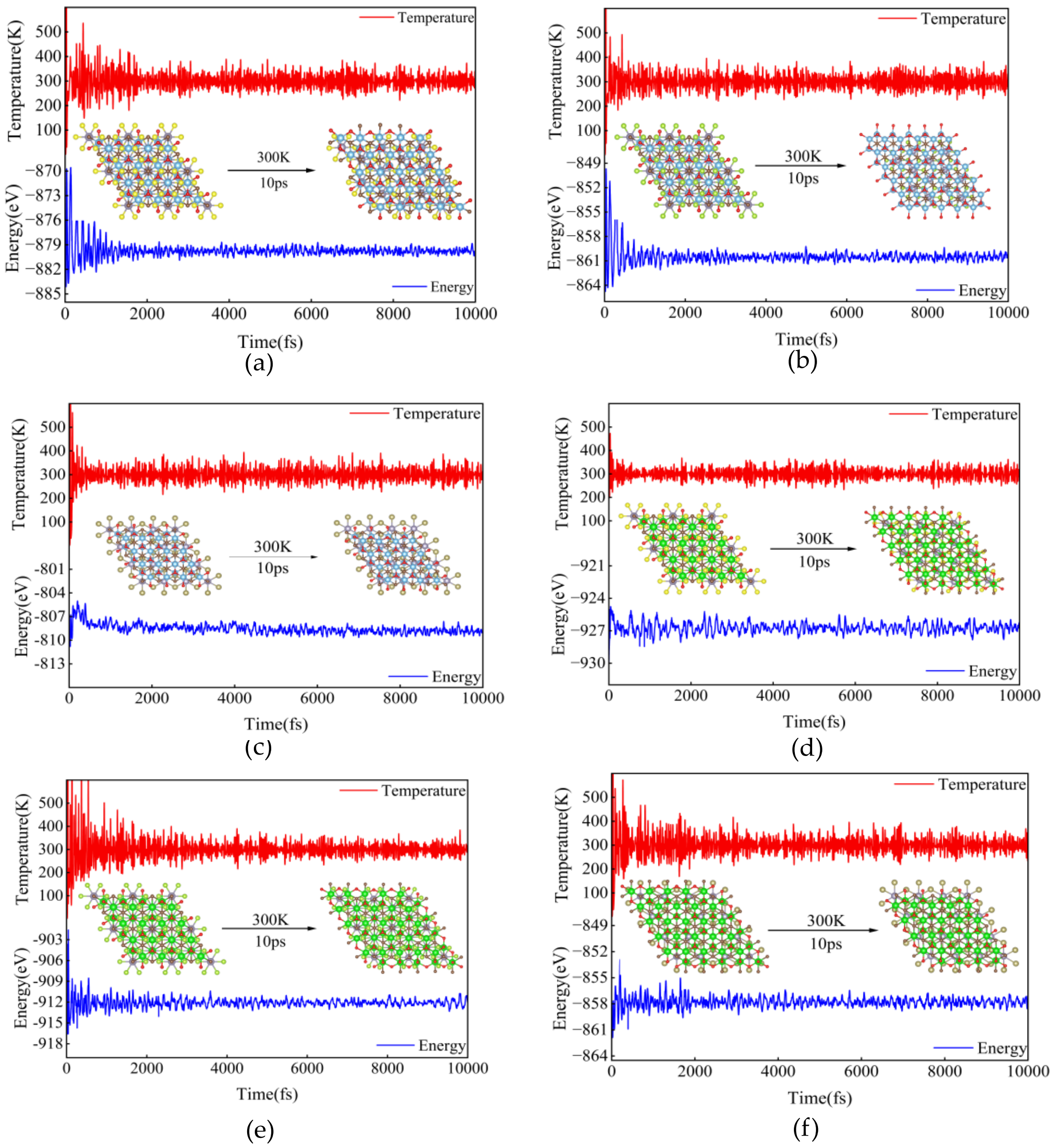

Fig S2 The ab initio molecular dynamics (AIMD) simulates the energy and temperature for (a)SnS_2/Ti_2CO_2, (b)$SnSe_2/Ti_2CO_2$, (c)$SnTe_2/Ti_2CO_2$, (d)SnS_2/Zr_2CO_2, (e)$SnSe_2/Zr_2CO_2$, and (f)$SnTe_2/Zr_2CO_2$.

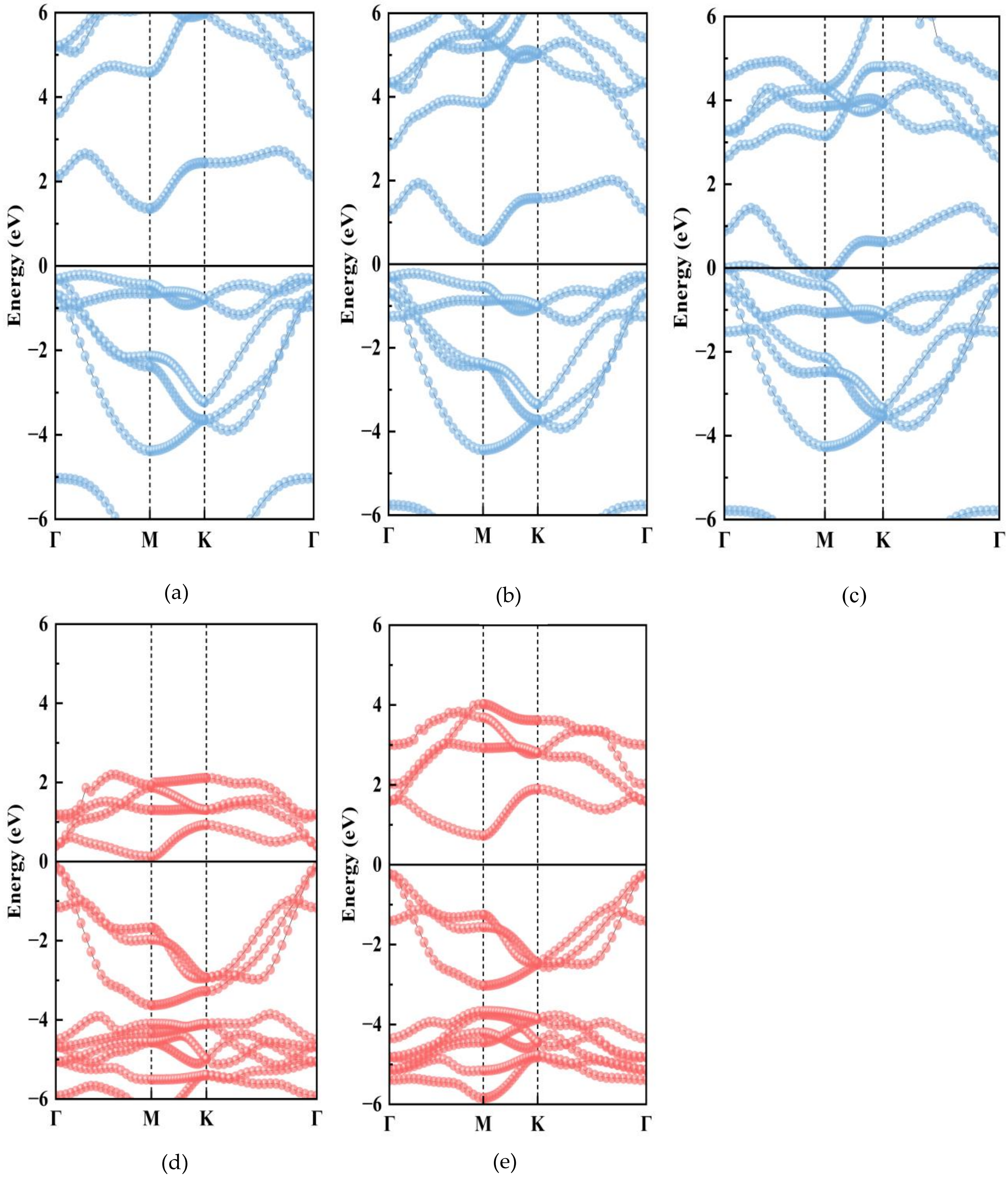

Fig S3 The computed band structures of (a) SnS_2, (b) $SnSe_2$, (c) $SnTe_2$, (d) Ti_2CO_2, and (e) Zr_2CO_2. The Fermi level was set to zero.

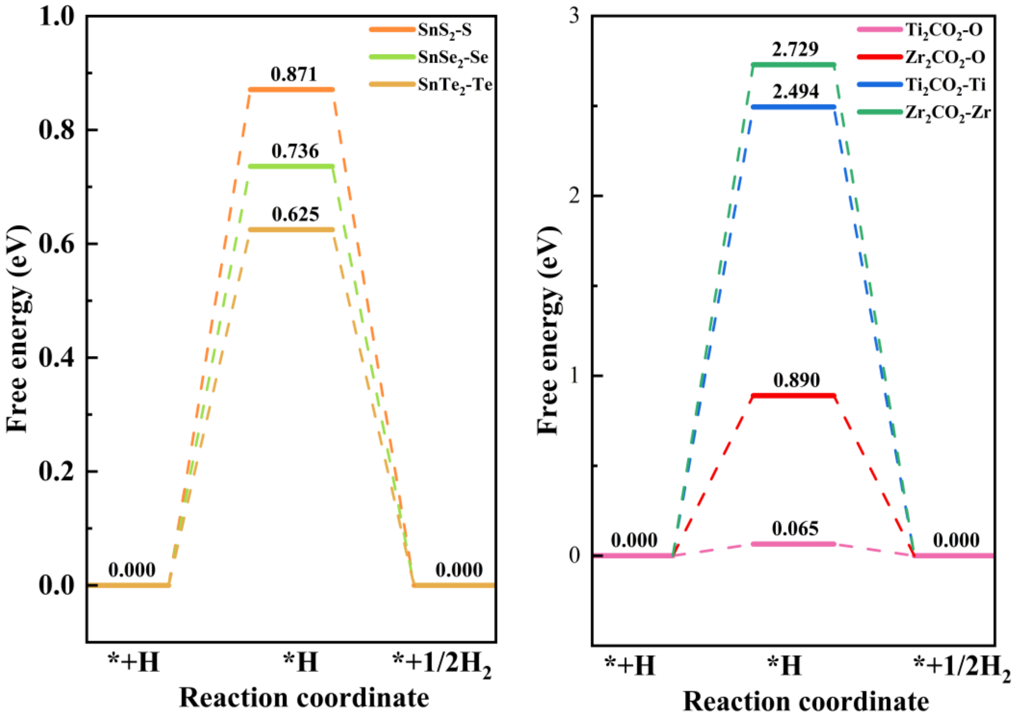

(a) (b)

Fig S4 The HER performance of (a) SnS_2, $SnSe_2$, $SnTe_2$, and (b) Ti_2CO_2, Zr_2CO_2.

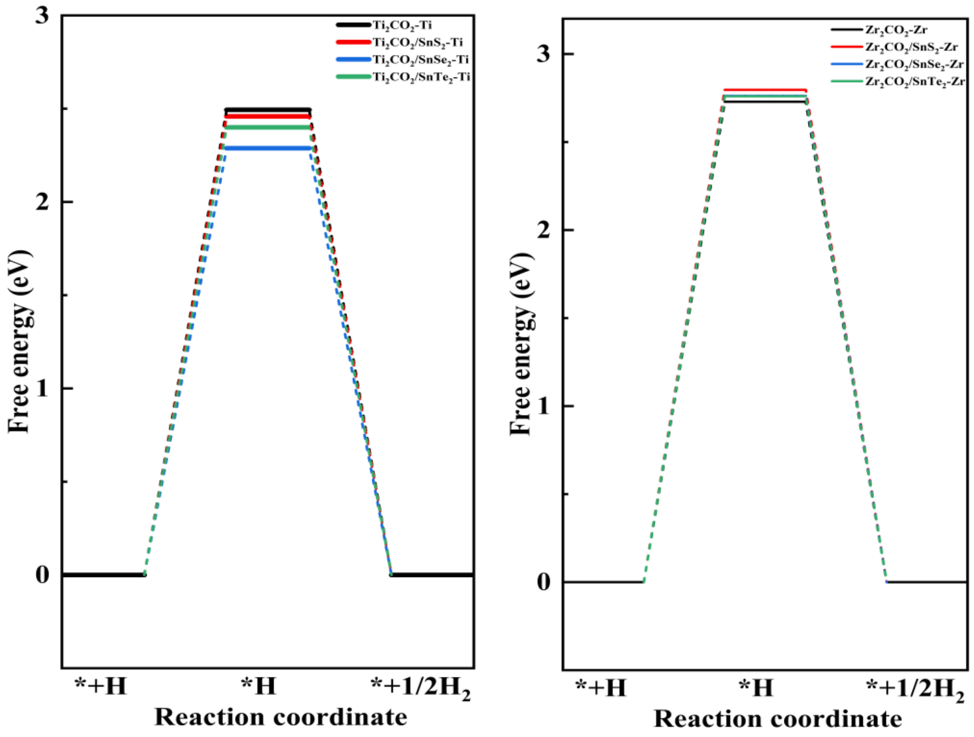

(a) (b)

Fig S5 The HER performance change of (a) Ti site on Ti_2CO_2, (b)Zr site on Zr_2CO_2.

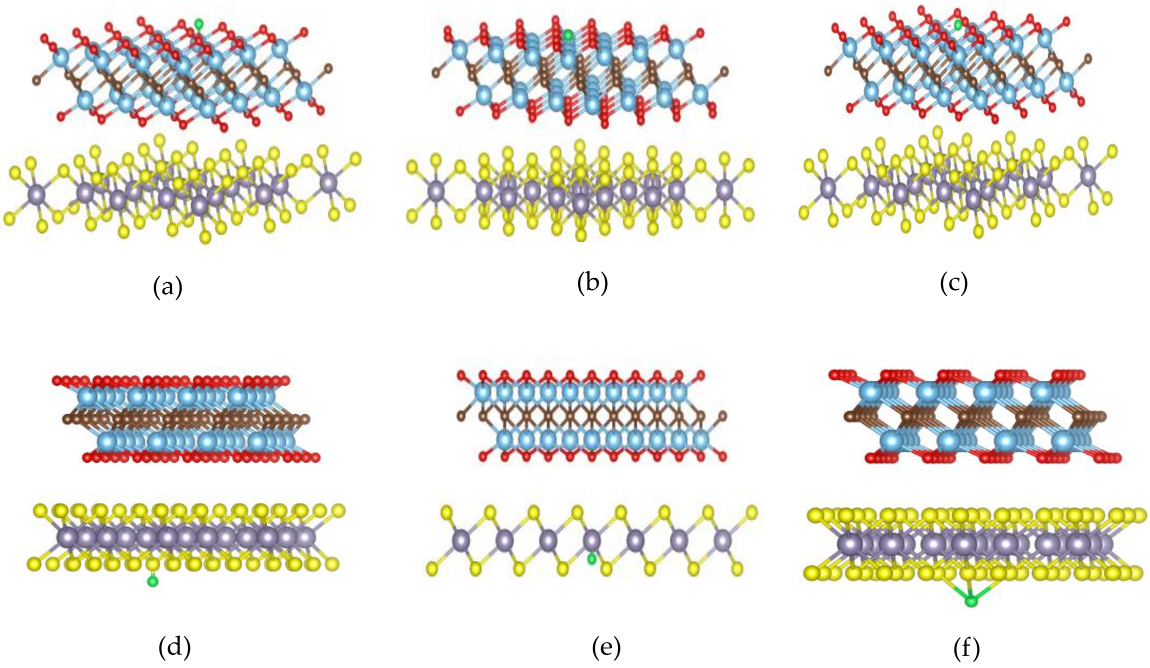

Fig S6 Various possible adsorption sites (a) O site on Ti_2CO_2, (b) Ti site on Ti_2CO_2, (c) Hollow site on Ti_2CO_2, (d) S site on SnS_2, (e) Sn site on SnS_2, (f) Hollow site on SnS_2. Hydrogen atoms are shown in green.

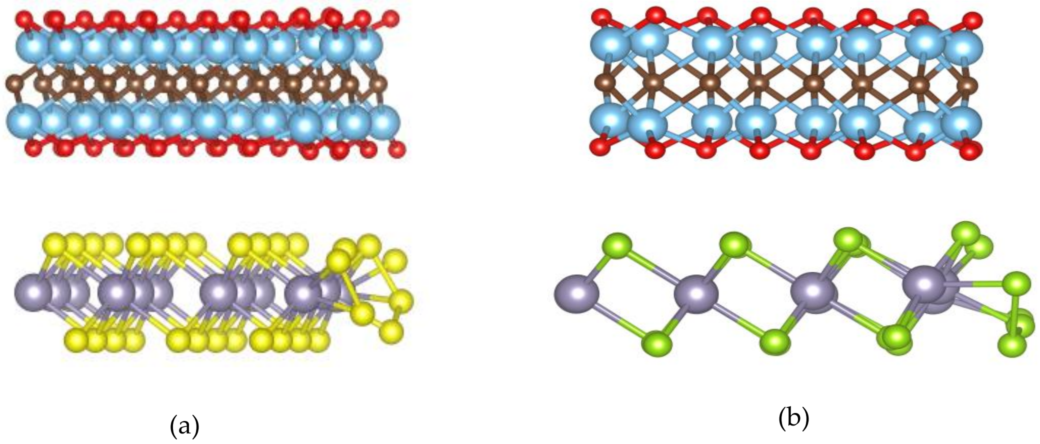

Fig S7 Optimization of active sites at the heterostructure interface of (a) SnS_2/Ti_2CO_2, (b) $SnSe_2/Ti_2CO_2$.

References

1. Chen, Y.; Meng, G.; Yang, T.; Chen, C.; Chang, Z.; Kong, F.; Tian, H.; Cui, X.; Hou, X.; Shi, J. Interfacial Engineering of Co-Doped 1T-MoS2 Coupled with V2C MXene for Efficient Electrocatalytic Hydrogen Evolution. *Chem. Eng. J.* **2022**, *450*, 138157.
2. Asad, M.; Rasheed, L.; Habib, H.; Miao, B.; Younas, A.; Majeed, S.; Anwar, M.I.; Meer, I.A. Synergistic Interface Engineering of Transition Metal-Doped ReS2/Ti3C2 MXene Hybrid for Boosted Electrocatalytic HER: Experimental and DFT Insights. *J. Power Sources* **2026**, *673*, 239724.
3. Wu, Y.; Zhang, Z.; Peng, Y.; Huang, B.; Zhou, N.; Li, N. TMDs-Passivated MXenes as Boosting Electrocatalyst for Hydrogen Evolution Reaction. *J. Solid State Chem.* **2024**, *331*, 124530.
4. Hu, J.; Liu, X.; Wang, J.; Jin, J.; Ouyang, M.; Fan, M.; Zhang, R.; Miao, L.; Jiang, J. Asymmetrical Zr2CO/VSe2 Heterostructure as Efficient Electrocatalysts for Hydrogen Evolution Reaction., doi:10.1039/D4NJ00906A.

Disclaimer/Publisher's Note: The statements, opinions and data contained in all publications are solely those of the individual author(s) and contributor(s) and not of MDPI and/or the editor(s). MDPI and/or the editor(s) disclaim responsibility for any injury to people or property resulting from any ideas, methods, instructions or products referred to in the content.
